# Beyond difficulties in self-regulation: the role of identity integration and personality functioning in young women with disordered eating behaviours

**DOI:** 10.1186/s40337-021-00398-5

**Published:** 2021-07-31

**Authors:** Marko Biberdzic, Josephine Tang, Junhao Tan

**Affiliations:** School of Psychology, University of Wollongong, Wollongong, NSW 2522 Australia

**Keywords:** Disordered eating behaviours, Identity integration, Personality functioning, Eating disorders, Self-regulation

## Abstract

**Background:**

Past research has established individual relationships between disordered eating behaviours (DEB) and both self-regulation difficulties and identity disturbance. However, no research has looked at the shared influence of these constructs on DEB nor at personality functioning in individuals with DEB.

**Methods:**

In the present study, self-regulation was explored in terms of effortful control, impulsivity and emotion regulation while identity integration was measured in terms of impairments in self-functioning using a sample of 247 undergraduate students.

**Results:**

Significant associations were found between all components of self-regulation and DEB, with the exception of impulsivity. Identity instability was also associated with self-regulation difficulties and DEB. Structural Equation Modelling analyses indicated that identity instability partially mediated the relationship between self-regulation and DEB. Lastly, disordered eating was associated with difficulties in personality functioning, with young women presenting with DEB reporting significantly greater difficulties in both self and interpersonal personality functioning.

**Conclusion:**

Behavioural eating anomalies should be considered as epiphenomena secondary to a possible deeper issue that reflects difficulties related to identity integration and potential personality functioning. The clinical implications of these findings are discussed.

## Plain English summary

Eating disorders involve patterns of problematic eating and behaviours aimed at reducing body weight and/or preventing weight gain. Since early disordered eating behaviours such as dietary restraint and binge-eating are highly predictive of a later development of eating disorders, it is important to investigate the roots of these behaviours. This study aimed to compare different contributing factors, previously suggested to play a role in disordered eating behaviors, such as emotion regulation, impulsivity, and identity disturbance. We also aimed to examine the unique role of identity disturbance in explaining the relation between difficulties in emotion regulation and disordered eating behaviours. By using different questionnaires and a sample of 218 university women, we found that both emotion regulation and identity instability predicted disordered eating behaviours. However, part of the impact of poor emotion regulation was explained by identity disturbance, which suggests that the latter plays a more central role in the development of disordered eating behaviours. Our results therefore suggest that young women who present with disordered eating may have underlying difficulties related to their sense of identity, i.e. a more unstable sense of self, and may benefit from targeting this specific area in psychotherapy.

Eating disorders and disordered eating behaviours are estimated to affect over 16% of the Australian population [36], and are one of the most prevalent disorders in youth [24]. Although the aetiology of eating pathology is complex [13], it has been argued that several variables involved in self-regulation and associated inhibitory processes – such as impulsivity [52], emotion regulation [38] and effortful control [11] – play an important role in the manifestation of these behaviours. More recently, an increased amount of literature has also re-emphasized the role of identity impairment in the development of disturbed eating, with an unclear sense of self being considered as the main underlying factor and contributor to eating disorder symptomatology [51]. However, despite general consensus in the theoretical literature, very few studies have empirically tested the role of identity pathology as a pathway to disordered eating, and no study has investigated the combined relation between impulsivity, emotion regulation, effortful control and identity disturbance in predicting disordered eating behaviours.

### Eating disorders and disordered eating behaviours

Eating disorders (EDs) are characterized by continuous patterns of problematic eating and engagement in behaviours aimed at reducing body weight and/or preventing weight gain [1]. Disordered eating can include behaviours that reflect many but not all of the symptoms of feeding and eating disorders [1]. These disordered eating behaviours (DEB) are the most common indicators of the development of an eating disorder, with disordered eating being linked to a reduced ability to cope with stressful situations, as well as an increased incidence of suicidal thoughts and behaviours, particularly in youth [36]. Both eating disorders and DEB involve considerable psychological impairment and distress, and are associated with a range of serious medical complications [36]. The mortality rate for people with eating disorders is also one of the highest among all psychiatric illnesses [3], with these difficulties considered to be a serious public health concern [42].

### Self-regulation: emotion regulation, effortful control and impulsivity

While self-regulation is known to be a multi-dimensional construct, it is broadly characterised as the ability to have emotional, behavioural and cognitive control over contextual demands. Self-regulation also encompasses a whole range of terms and functions including behavioural inhibition, impulsivity, effortful control, emotion control and cognitive control [5]. These regulatory mechanisms allow the individual to make relevant responses through processes such as initiation, adjustment, interruption or inhibition of thoughts and behaviours [4]. Effective self-regulation is reflected in appropriate management of situations and impulses and is related to beneficial outcomes in all age groups [7]. Conversely, failure to self-regulate is associated with a range of unfavourable outcomes including the development of many psychological disorders.

In the context of eating disorders, it has been suggested that DEB such as dietary restraint and purging/binging are used to regulate emotion [43]. More specifically, in the affect regulation model, DEB is understood as a coping and compensatory strategy for temporary reduction of negative affect or increase in positive affect [30]. This in turn is believed to reinforce poor self-regulation as the binges reduce the intensity of the negative affect, making it more difficult to regulate eating impulses. For individuals in the restrictive subtype, it has been postulated that diet restraint can help in affect regulation by using hunger to temporarily decrease one’s reactivity towards emotional stimuli ([19]; for a review of the affect regulation model and escape theory, see [22, 23]). A recent meta-analysis by Prefit et al. [38] also reported medium-to-large effect sizes for the associations between maladaptive emotion regulation and EDs and eating-related symptoms. Emotion regulation difficulties did not differ across EDs, further supporting the transdiagnostic character of emotion regulation problems in eating pathology.

Numerous studies on eating disorders have also underlined the contribution of temperamental factors involved in self-regulation, especially effortful control (e.g., [10]). Effortful control is described by Rothbart and Bates [40] as the ‘capacity to inhibit dominant responses so as to activate sub-dominant ones’, and thus pertains to the ability to wilfully inhibit, activate, or change attention and behaviour. The capacity for effortful control has been linked with the modulation of emotional reactivity and behaviour in general [7], and has been identified more specifically as an important risk factor for eating disorders [50]. Associations between DEB and low effortful control have been consistently reported in both non-clinical [10] and clinical [11] samples.

Similarly, several studies have shown associations between DEB and impulsivity (e.g. [41]). Although clinically associated with the presence of binge eating, impulsivity and impulsive behaviour are not uncommon among other ED subtypes, including restricting anorexia nervosa [18], with impulsivity being more recently considered as a transdiagnostic characteristic of individuals with EDs [31]. Considering that impulsivity in EDs has also been associated with poorer prognosis [33], the importance of further investigating the role of impulsivity in the development and onset of DEB has recently been called upon.

### Identity integration and sense of self

Identity consolidation is not only a crucial developmental milestone in youth, but also a salient challenge experienced by many patients with EDs [51]. The association between identity pathology and disordered eating has its roots in early psychodynamic thinking where EDs have traditionally been characterized as disorders of the self [8].

Bruch [9], among others, argued that identity impairment was the main predictor of EDs. Limited opportunities for autonomous functioning in childhood were posited to interfere with the development of a clear and coherent sense of self, which in turn contributed to feelings of incompetency, self-doubt and fear of losing control [8]. In concurrence with the corresponding feeling of powerlessness, an unstable sense of self is argued to lead to body weight and eating behaviours which are salient and more controllable [9]. Engagement in DEB would thus provide short-term relief from more pervasive and intolerable inner experiences.

Furthermore, Bruch [8] observed that patients with anorexia manifest difficulties in accurately perceiving or interpreting stimuli arising in their bodies, such as hunger and satiety, as well as often being unable to describe their emotions. She argued that one’s lack of awareness of inner experiences and failure to rely on feelings, thoughts, and bodily sensations to guide behaviour, may contribute to an overwhelming sense of ineffectiveness and an overall lack of agency [8]. The DEBs that follow could therefore be understood as the patient’s effort to compensate for these underlying deficits.

Consistent with the theory, several studies have investigated the association between dimensions of the self-concept (including global self-esteem, unclear sense of self, attitudes toward body image) and ED symptomatology. For example, individuals with bulimia were shown to have greater identity disturbance than controls as reflected through greater confusion, enmeshment and overall instability of sense of self [43]. These results were found to be consistent with the findings of Stein and Corte [48] who reported overall identity disturbances in women with AN and BN. More recently, similar difficulties with identity integration in a clinical sample of women with EDs were also associated with borderline personality symptomatology [51].

### Identity consolidation as an indicator of personality functioning

Considering that identity disturbance is considered to be a core indicator of personality pathology as defined in the DSM-5’s Alternative Model of Personality Disorders ([AMPD], [1]), the high prevalence and comorbidity of personality disorders in individuals with EDs should not come as a surprise (for a review see [34]). On the contrary, disturbance in personality functioning has been found to contribute to poor treatment outcome and to contribute to the persistence of ED symptomatology in some patients [16]. Nonetheless, although research has established a link between eating pathology and identity problems, and despite many suggesting that identity disturbance may contribute to ED development [54], few studies have empirically investigated the role of identity pathology as a potential pathway to DEB.

### Present study

Since early DEBs such as dietary restraint, bingeing and purging are highly predictive of a later onset of EDs, it is imperative to investigate the contributing factors of these behaviours and their potential associations with other indicators of psychopathology. Known for its multifactorial origin, extensive research has been done on the etiology of DEB [39]. In particular, psychological factors involved in self-regulation – namely emotion regulation [32], effortful control [12] and impulsivity [31] – and impairments in identity and self-functioning [51] have consistently been associated with ED symptomatology. While extensive research has been done on the individual contributions of these variables, the existing study is the first to investigate the role of identity disturbance in explaining the relation between core deficits in self-regulation and DEBs. In addition, the current study also aimed to investigate impairments in personality functioning in individuals presenting with DEB. It was hypothesized that: 1) poor self-regulation, as defined by greater difficulties with emotion regulation, impulsivity and effortful control, would be associated with higher occurrences of DEB; 2) identity disturbance would mediate the relationship between self-regulation and DEB; and 3) individuals presenting with DEB would have greater impairments in personality functioning than those not engaging in disordered eating.

## Methods

### Participants and procedure

A total of 247 undergraduate psychology students from the University of Wollongong were initially recruited for this study, with 28 males (11.3%) and 218 females (88.7%). Considering the significantly uneven distribution and poor representation of males, only the female population was included in the final sample. Participants’ age ranged from 18 to 25 years old (*M* = 19.39, *SD* = 1.70). Amongst these, 94 participants (43.1%) had a mental health diagnosis while nearly half of the total sample reported a mental health diagnosis in their immediate family members (107 participants; 49.1%). These mental health diagnoses included depressive disorders, anxiety disorders, eating disorders, bipolar I and II disorder, schizoaffective disorder, obsessive-compulsive disorder, post-traumatic stress disorder, and borderline personality disorder. Participation was voluntary and consent was obtained through the consent form after reviewing the study’s information found in the participant information sheet. As we were interested in assessing DEB in youth, participants were required to be of age 18 to 25 years old and proficient in English. Upon giving their consent, participants completed the demographic information sheet before proceeding on to complete the self-report measures described below. Participants took on average about 20 min to complete the survey and were awarded 0.5 credit for research participation. Items within the survey were presented in random order and were not part of a larger survey battery.

### Measures

#### Adult temperament questionnaire (ATQ)

The ATQ-short form [15] consists of a total of four scales, including Negative Affect, Extraversion/Surgency, Effortful Control and Orienting Sensitivity. Only the Effortful Control scale of the ATQ-short form was used in the present study. The scale comprises 19 items to be rated in a 7-point Likert scale (1 = *extremely untrue of you*; 7 = *extremely true of you*) and is divided into three subscales: inhibitory control (i.e. capacity to inhibit inappropriate behaviour; 7 items), attentional control (i.e. capacity to focus attention as well as to shift attention when desired; 5 items) and activation control (i.e. capacity to perform an action when there is a strong tendency to avoid it; 7 items). Internal consistency for the Effortful Control scales was adequate in the current study (*α*= .75).

#### Barratt impulsiveness scale-15 (BIS-15)

The BIS-15 [45] is a brief version of one of the most commonly used scales to assess impulsivity. Similar to its predecessor, the BIS-11 [37], this instrument assessed overall impulsivity across three domains: non-planning, motor, and attention impulsivity. Participants respond on a 4-point Likert scale. Within the current study, these three subscales indicated adequate reliabilities of *α* = .71, *α* = .79, and *α* = .74 respectively.

#### Severity indices of personality problems-118 (SIPP-118)

The SIPP-118 is a self-report questionnaire that assesses the core components of (mal) adaptive personality functioning. It contains 118 items that are answered on a 4-point Likert scale, and cover 5 domains including 16 facets of personality functioning. Only the Identity Integration domain and Emotional Regulation facet of the SIPP-118 were used in this study. The Emotional regulation facet measures the capacity for emotional tolerance and control, whereas the Identity Integration domain encompasses five facets reflecting an integrated sense of self, including: Self-respect, Stable self-image, Self-reflexive functioning, Enjoyment and Purposefulness. Both Identity Integration and Emotional regulation indicated adequate reliabilities of *α* = .94 and *α* = .74 respectively.

#### Levels of personality functioning scale-brief form 2.0 (LPFS-BF 2.0)

The LPFS-BF 2.0 [53] is a brief self-report questionnaire, which assesses the LPFS as described in Section III of the DSM-5 [1]. The LPFS-BF 2.0 consists of 12 items, clustered into two higher order domains: self-functioning and interpersonal functioning. Participants are asked to rate the 12 items on a 4-point Likert scale from 1 (completely untrue) to 4 (completely true). Both domains had satisfactory reliability in this study (Self-functioning *α* = .82; Interpersonal funtioning *α* = .74).

#### Eating disorder examination questionnaire 6.0 (EDE-Q 6.0)

The EDE-Q 6.0 was designed to assess the key behaviour-oriented aspects of eating disorders over the past 4 weeks (28 days) and consists of four main subscales (Restraint, Eating concern, Shape concern and Weight concern). Frequencies relating to DEB such as binging and purging were also assessed with items surrounding overeating and attempts at losing weight with extreme measures. Participants responded on a 7-point Likert scales that differed depending on the question (e.g. 1 ‘No days’ to 7 ‘Every day’). All individual subscales had good to excellent reliabilities (*α* = .84 for restraint, *α* = .84 for eating concern, *α* = .92 for shape concern and *α* = .88 for weight concern). According to the DSM-5 [1], binge eating and inappropriate compensatory behaviours both occur, on average, at least once a week for 3 months for individuals diagnosed with Bulimia Nervosa. In order to distinguish individuals presenting with DEB from those without, the presence of DEB was defined as individuals with at least four occurrences of either binge eating or inappropriate compensatory behaviours or both over the last month.

### Data analysis strategy

The Statistical Package for Social Sciences (SPSS Version 25.0; [26]) software and Amos [2] were used in the present study for all analyses including regression and structural equation modelling (SEM). A bivariate correlations analysis was conducted between effortful control, emotional regulation, impulsivity, identity integration, personality functioning, and DEB. Following that, the current study examined the mediating effect of identity integration and personality functioning in the relationship between self-regulation and DEB in two separate SEM analyses. Based on the recommendation by Shrout and Bolger [44], mediation was examined via bootstrapping, by estimating the confidence intervals of the indirect effects to test their significance. Finally, by using the six behavioural items of the EDE-Q, personality functioning scores of participants with presence of DEB was compared with those without.

## Results

### Preliminary analyses

Preliminary analyses were conducted to determine the confounding effects of age and previous history of mental health conditions on the variables of interest. No effects of age were found through correlational analysis. However, young women with a prior history of mental health conditions scored significantly higher on all variables of interest when compared with those without a previous history. As such, history of mental health conditions was controlled for.

Bivariate Pearson’s correlation coefficients were calculated to test for the direction and strength of the linear relationships between all individual components of the main variables as shown in Table 1. Results showed DEB to significantly and positively correlate with effortful control, emotional regulation, identity integration, and personality functioning, but not impulsivity. Out of the four subscales of the EDE-Q, eating, shape, and weight concerns also correlated significantly with effortful control, emotional regulation, identity integration, and personality functioning. Notably, restrain correlated with only identity integration and personality functioning. Due to impulsivity’s lack of significant associations with other variables, it was excluded from further analyses.

**Table 1:** Correlations between main variables

	1	2	3	4	5	6	7	8	9	10	11	12
1. Effortful Control-ATQ	–											
2. Emotional Regulation-SIPP	.407**	–										
3. Impulsivity-BIS-15	.400**	.348**	–									
4. Identity Integration-SIPP	.427**	.598**	.363**	–								
5. Personality Functioning-LPFS	.456**	.592**	.398**	.836**	–							
6. Restraint-EDE-Q 6.0	.100	.023	.106	.220**	.175*	–						
7. Eating Concerns-EDE-Q 6.0	.201*	.150*	.080	.471**	.354**	.639**	–					
9. Shape Concerns-EDE-Q 6.0	.150*	.164*	.111	.443**	.335**	.642**	.746**	–				
9. Weight Concerns-EDE-Q 6.0	.165*	.149*	.100	.423**	.317**	.642**	.732**	.939**	–			
10. Global Score-EDE-Q 6.0	.171*	.147*	.111	.448**	.339**	.775**	.856**	.963**	.951**	–		
11. Binging behaviours	.173*	.143*	.038	.264**	.230**	.273**	.477**	.388**	.362**	.419**	–	
12. Purging behaviours	.009	.018	−.001	.130	.134	.517**	.526**	.397**	.418**	.495**	.152*	–

*N* = 218

*ATQ* Adult Temperament Questionnaire, *SIPP* Severity Indices of Personality Problems-118, *BIS-15* Barratt Impulsiveness Scales-15, *LPFS* Levels of Personality Functioning Scale-Brief Form 2.0, *EDE-Q 6.0* Eating Disorder Examination-Questionnaire 6.0

* = *p* < .05, ** = *p* < .001

### Structural equation modelling

**Fig. 1:**
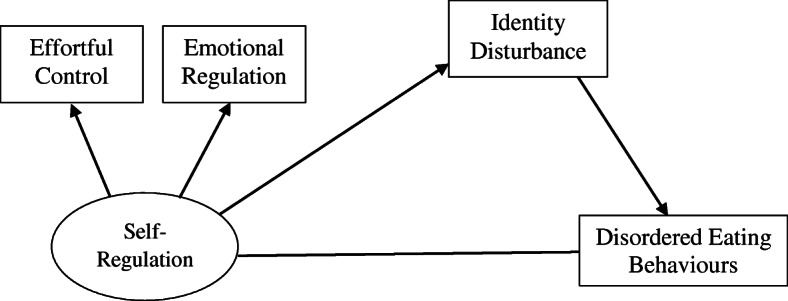
Mediation model with main variables of interest. Note. Confounding variable (history of mental health) was not included in the figure

SEM analyses were conducted to investigate the mediating effect of identity integration on the relationship between self-regulation and DEB as illustrated in Fig. 1. All analyses were done with 5000 bootstrapped samples with a 95% confidence interval for each indirect effect. We challenged the obtained results by running a second mediation model using personality functioning as the mediating variable. Results for both of these analyses are presented in Tables 2 and 3.

**Table 2:** Path standardized coefficients and indirect effects for identity integration as mediator

	Path Coefficients		Estimate	Indirect Effects	
	to Identity Integration	to Disordered Eating Behaviours		95% Confidence Interval	
				Lower	Upper
Mental Health Hx	.038	.129			
Self-Regulation	.780***	−.374*			
Identity Integration		.711***			
SR → II → DEB			.555***	1.563	5.037

*N =* 246

*Mental Health Hx* Mental Health History, *SR* Self-Regulation, *II* Identity Integration, *DEB* Disordered Eating Behaviours

* = *p* < .05, *** = *p* < .001

**Table 3:** Path standardized coefficient and indirect effects for personality functioning as mediator

	Path Coefficient			Estimate	Indirect Effects	
	to Self-Functioning	to Interpersonal Functioning	to Disordered Eating Behaviour		95% Confidence Interval	
					Lower	Upper
Mental Health Hx	−.009	.013	.149*			
Self-Regulation	.772***	.703***	−.201			
Self-Functioning			.450**			
Interpersonal Functioning			.076			
SR → SF → DEB				.348***	.671	2.906
SR → IF → DEB				.053	−.315	.495

*N =* 246

*Mental Health Hx* Prior history of Mental Health Conditions, *SR* Self-Regulation, *SF* Self-Functioning, *IF* Interpersonal-Functioning, *DEB* Disordered Eating Behaviours

* = *p* < .05, ** = *p* < .01, *** = *p* < .001

In the first mediation model, modelling prior mental health conditions as a confounding variable showed that it is not significantly related to both DEB and identity integration. Further results showed that self-regulation had significant indirect effects on DEB through identity integration (*β* = .555, *p* < .001 : CI = [1.563, 5.037]). Hence, results indicated a mediating effect of identity integration on the relationship between DEB and self-regulation as measured by both emotional regulation and effortful control. Furthermore, since the resulting direct effects between both components of self-regulation and DEB were significant, results suggest a partial mediating effect of identity integration. Per Kline [27] and Hooper et al. [25], the recommended cut-offs of goodness of fit indices indicative of a good model fit are as follow: *χ*^2^ *p* > .05, CFI > .90, CFI > .95, NFI > .95, RMSEA < .08, SRMR < .08. The goodness of fit indices (*χ*^2^(2) = 2.816, *p* = .245, CFI = .996, GFI = .995, NFI = .987, RMSEA = .043, SRMR = .024) suggest an excellent fit between the observed data and the current model.

When looking more broadly at personality functioning as the mediating variable, self-regulation was shown to have significant indirect effects on DEB through self-functioning (*β* = .348, *p* < .001 : CI = [.671, 2.906]). However, interpersonal functioning did not have any significant effect on DEB (*β* = .076, *p* > .05). Because the resulting direct effects between self-regulation and DEB were nonsignificant, results suggest a complete mediating effect (full mediation). Contrary to the first analysis, prior history of mental health conditions as a confounder had a significant relationship with DEB (*β* = .149, *p* < .05), but not with the other two mediators. Similar to the first model, fit indices suggested excellent fit: *χ*^2^(4) = 4.010, *p* = .405, CFI = .999, GFI = .994, NFI = .986, RMSEA = .003, SRMR = .019.

### Independent samples *T*-test

Finally, independent samples *t-*tests were conducted to investigate whether young women presenting with DEB would have greater impairments in personality functioning compared to those not engaging in disordered eating (see Table 4). Results show that there were significant differences between the two groups on both self- and interpersonal-functioning scores. Specifically, young women who presented with DEB scored significantly higher on both self- (*t*(216) = 3.464, *p* < .01, *d =* .474) and interpersonal functioning (*t*(216) = 2.924, *p* < .01, *d =* .397) impairments than their counterparts, both with small to medium effect sizes.

**Table 4:** Comparing individuals with and without presence of DEB on personality functioning

Variable	DEB Presence	*N*	*M*	*SD*	*t*	*P*	Cohen’s D
Self-Functioning	Y	91	14.517	4.031	3.464	.001	.474
	N	127	12.638	3.889			
Interpersonal Functioning	Y	91	12.506	3.573	2.924	.004	.397
	N	127	11.181	3.087			

*N =* 218

## Discussion

The present study aimed to investigate the shared contribution of difficulties in self-regulation and identity disturbance to DEB. More specifically, this study aimed to investigate the role of identity instability as a mediator of the relationship between effortful control, impulsivity, emotion regulation and DEB. Significant associations were found between all components of self-regulation and DEB, with the exception of impulsivity. Both identity instability and personality dysfunction were also associated with DEB. Binging behaviours more specifically were associated with effortful control deficits, emotional regulation difficulties, identity instability and personality dysfunction. Eating restraint was however only associated with identity instability and personality dysfunction. Moreover, findings suggest that the effect of both emotion regulation and effortful control on DEB was non-significant once identity instability was introduced in the model, as it fully mediated the relationship between both variables and DEB.

The significant association between disordered eating and both poor effortful control and emotion regulation difficulties are consistent with results from previous studies that have highlighted the role of these variables in the manifestation of DEB (e.g. [30]). However, these associations were not as strong as those between DEB and difficulties in identity integration and personality functioning. In fact, our findings suggest that difficulties in both effortful control and emotion regulation only impact disordered eating through the effect of identity disturbance. These results were further supported when comparing participants who engaged in DEB with those who did not, with the former group displaying significantly more difficulties in both self and interpersonal domains of personality functioning. However, difficulties in interpersonal functioning were not significant predictors of DEB.

Most importantly, our findings suggest that behavioural eating anomalies may best be understood as epiphenomena secondary to a potentially deeper psychopathological core that reflects difficulties related to identity integration and personality functioning. This is in line with findings from Stanghellini et al. [46] who found that the central psychopathological features of shape and weight concerns in EDs are the result of a disturbance in the way these individuals experience their own body and difficulties in defining their identity. From a developmental perspective, the body plays an important role in the process of identity development and integration, and self-organization initially entails the integration of body-related experiences [49]. It is therefore not surprising that individuals with ED pathology have difficulty recognizing and making sense of internal bodily sensations as this reflects the basic form of self-awareness [47].

Although emotional regulation difficulties were included in our model as preceding identity integration, we acknowledge the bi-directional relationship between these two variables, and that both may reinforce each other through a vicious circle. For example, it is possible that difficulties with affect regulation may be secondary to unclear and unstable representations of self in individuals who engage in DEB, and that individuals who have difficulty making sense of their internal world may resort to behaviours like bingeing and purging to decrease the intensity of negative emotional states. As previously suggested, DEB can thus be understood as specific strategies of affect regulation [43], linked to an inability to contain, metabolize and mentalize affects. Likewise, as argued by many, DEB could also represent attempts to “take care of one’s self, a search for a sense of subjectivity and interpersonal efficacy through the control of eating behaviours” ([21], p.53).

Lastly, our findings related to the potential difficulties in personality functioning in young women who present with DEB echo the high resistance to treatment that has been reported in patients with EDs [17]. Indeed, research has shown that the treatment of EDs is often of modest effect and that significant difficulties persist after treatment [29]. More specifically, existing studies show that personality pathology is one of the most comorbid presentations in EDs [14]. As a recent meta-analysis of 87 studies has shown, more than half of ED patients have comorbid PD diagnoses [34]. Individuals with EDs also often present with ego-syntonic features and a chronic course of illness occurs in a considerable number of these individuals, as is the case in individuals with PDs [28]. It is therefore possible that variance in the onset, clinical course, symptomatic profile, and maintenance of ED symptomatology can be explained by underlying difficulties related to personality functioning and identity integration more specifically. The importance of considering personality functioning more broadly was also further supported by the significant differences observed between young women presenting with DEB and those without on the LPFS (despite interpersonal functioning not being a significant mediator in the tested models). Our findings thus support the argument that EDs should be considered as developmental disorders [17] and difficulties around identity integration and personality functioning as crucial issues involved in treatment resistance.

Our findings should however be interpreted in light of certain limitations. First, all analyses were cross-sectional and therefore, causal relationships cannot be directly inferred. Although the use of SEM has been found to have sound psychometric value in comparison to simple mediation analysis (see [6]), fitting the data does not “prove” the causal assumptions and a strong mediator in a cross-sectional analysis is not guaranteed to be a significant mediator in a longitudinal design [35]”. Second, our sample consisted of self-selected, female psychology students and was relatively small. In addition, the racial breakdown of our participants was unknown. Although this proportion is to be expected considering that DEB and EDs are more prevalent in women [20], future studies that include a larger, male, and more age-diverse sample are needed to ensure generalizability of the results and assess the roles of ethnicity in these models. Lastly, the measures used in this study were exclusively self-reports. It would have been desirable to have access to structured clinical interview data, especially considering the ego-syntonic nature of these difficulties. Future studies should therefore look to replicate these findings in clinical populations using a longitudinal framework as well as investigate how some of the well-documented etiological factors (e.g. abuse and negative family experiences) may specifically relate to the development of identity disturbance and personality functioning in individuals with EDs.

## Conclusion

Taken together, our results support early theoretical understandings [8] and extend findings from recent empirical work [51] that suggest that eating disorders are essentially disorders of the *self*. Unfortunately, in the current bio-psycho-social paradigm, the focus remains directed at external factors such as body mass index, food behaviors, or weight and shape comparisons. Our findings support previously suggested models (e.g. [17]) where the profound nature and meaning underlying the symptomatology of these disorders should be prioritized over the previously mentioned surface-level behaviours. It is possible that another reason for the limited effectiveness of treatment in these patients, is in fact the extensive focus on DEB rather than on the underlying issues that underpin these behaviours and the frequent secondary gain associated with disordered eating symptomatology. Moreover, our findings highlight the importance of assessing personality (dys) function and identity disturbance when working with individuals who engage in DEB, considering the clinical implications of personality-related difficulties (e.g. greater resistance, negative transference, tenuous therapeutic relationship). Although patients with EDs may present at times with symptomatology so severe that it becomes tempting to focus solely on the maladaptive eating, clinicians should not forget the interpersonal nature of these difficulties and that the core of the disorder may still lie in difficulties related to identity integration with different etiological factors potentially interfering with healthy personality development, especially in youth.

## Data Availability

The datasets used and/or analysed during the current study are available from the corresponding author on reasonable request.

## Abbreviations

APA: American Psychological Association
AN-BP: Anorexia Nervosa – Binge/Purge
AN-R: Anorexia Nervosa – Restricting
ATQ: Adult Temperament Questionnaire
BED: Binge-Eating Disorder
BIS: Barratt Impulsiveness Scale
BN: Bulimia Nervosa
CFI: Comparative Fit Index
DEB: Disordered Eating Behaviour(s)
ED: Eating Disorder(s)
EDE-Q: Eating Disorder Examination Questionnaire
GFI: Goodness of Fit Index
LPFS: Level of Personality Functioning Scale
NEDC: National Eating Disorders Collaboration
NFI: Normed Fit Index
RMSEA: Root Mean Square Error of Approximation
SEM: Structural Equation Modelling
SIPP: Severity Indices of Personality Problems
